# Extracellular Vesicles – Powerful Markers of Cancer EVolution

**DOI:** 10.3389/fimmu.2014.00685

**Published:** 2015-01-12

**Authors:** Joana Carvalho, Carla Oliveira

**Affiliations:** ^1^Institute of Molecular Pathology and Immunology of University of Porto (IPATIMUP), Porto, Portugal; ^2^Department of Pathology and Oncology, Faculty of Medicine of University of Porto, Porto, Portugal

**Keywords:** extracellular vesicles, longitudinal studies, precision-medicine, cancer biomarkers, liquid biopsy

Solid tumors are likely derived from the co-evolution of neoplastic cells, stromal components, vasculature, and immune cells (1). As tumors evolve and progress, an array of molecular changes accumulate giving rise to multiple cell subpopulations, each with the ability to divide and mutate further. In addition, neoplastic cell populations are able to modulate the behavior of other types of cells in their microenvironment, converting their intrinsic anti-tumoral into pro-tumoral activity (2, 3). Therefore, a malignant tumor is composed not only of neoplastic cells that are heterogeneous in terms of genetic and phenotypic features, but also by different pro-tumoral cells and a particular extracellular matrix that supports cancer evolution and progression.

Tumor heterogeneity has been recognized as one of the main factors for cancer therapy failure, and has just started to be dissected using next-generation sequencing (NGS) approaches (4). While whole genome sequencing, and particularly, exome sequencing have provided the molecular basis for several complex traits, RNA and bisulfite sequencing have been important to disclose expression regulatory mechanisms. However, NGS-derived studies have often been conducted using single fragments/biopsies of primary tumors, and therefore fail to reflect the global tumor heterogeneity, dynamics, and drug sensitivities, likely to change during tumor evolution and treatment. For these reasons, there is the need to develop strategies that may accurately capture the entire landscape and allow following clonal evolution of tumor populations.

Scientific evidence supports that most types of cells secrete small vesicles (exosomes and microvesicles) into the extracellular milieu (5), and that tumor cells in particular produce at least threefold more of these small vesicles than normal cells (6–8). These so-called extracellular vesicles (EVs) are emerging mediators of intercellular communication and orchestrators of health and disease, and contain a repertoire of genetic information (incorporated in DNA, RNA, microRNAs, and proteins), which may be a fingerprint of the releasing cell type (9, 10). EVs can be easily detected in biological fluids such as plasma, serum, ascites, or urine, and provide excellent minimally invasive biomarker candidates to monitor cancer patients’ progression, prognosis, and treatment efficacy (10, 11). In fact, tumor-derived exosomes in patients’ bloodstream were shown to contain fractions of tumor genome, transcriptome, and proteome such as *KRAS, TP53* mutations in pancreatic and colon cancer (12); mutant/variant *EGFRvIII* mRNAs in glioblastoma (13); microRNAs in ovarian cancer (14); MET in melanoma (15); and HER2 in breast cancer (16). Further, double-stranded DNA (exoDNA) representing the entire genome and reflecting the mutational status of parental tumor cells [e.g., *BRAF*(V600E) and *EGFR* exon 19 deletion — del19], was found in EVs from melanoma and non-small cell lung cancer cell lines (17). From a clinical perspective, molecules enclosed in EVs harbor potential usefulness as circulating biomarkers with impact in early detection and during cancer progression. Apart from carrying specific molecular signatures and disease effectors, EVs also contribute to horizontal cellular transformation and phenotypic reprograming, both locally and systemically (8, 10, 15, 18).

The identification of specific EV features has allowed developing isolation and characterization methodologies that have been used in numerous studies (19–21). Most of these have been focused on the characterization of the cargo of EVs in different types of cancer, using either conditioned media of cancer cell lines, or unique samples from cancer patients’ body fluids. Cancer cell line studies have provided markers of EVs for different types of cancers, however, lack the representativeness of cancer as a heterogeneous cell population. Unique samples from cancer patients’ body fluids have highlighted potential markers for cancer diagnosis and prognosis in cross-sectional studies, although fail to deliver useful information to monitor tumor heterogeneity and dynamics, and to allow therapy response and recurrence assessment.

Longitudinal studies of cancer patients, from whom samples are repeatedly collected along diagnosis, treatment, and follow-up, have been rarely reported, and may be the most adequate tools to address the abovementioned limitations. One possible longitudinal approach should enclose the molecular profiling of the following patient-derived samples: (1) biopsy/surgical tumor specimens; (2) body fluid-derived EVs collected prior/at surgery; (3) body fluid-derived EVs collected immediately after surgery; (4) body fluid-derived EVs collected along therapy cycles, and; (5) body fluid-derived EVs collected after disease remission (if possible). The comparative analysis of data derived from each of these datasets will shed light into cancer-specific signatures that become represented in tumor-derived EVs (biomarker candidates), and that should be used to monitor tumor evolution, dynamics, and therapy response, as well as predict disease recurrence. This type of studies raises the need for close collaborations between clinical and basic research teams, to set-up effective study designs and ethically approved protocols, to allow collection of multiple samples and clinically relevant information from each individual patient. The power of this approach is the possibility of providing information useful for the design of precision-medicine approaches, with impact in clinical practice.

In summary, in this article, we discuss the impact of performing longitudinal studies through the analysis of EVs from cancer patients, to improve our understanding of tumor heterogeneity/evolution, and to identify minimally invasive markers, potentially useful for disease management of cancer patients. We further present a workflow that may be useful to consider when designing longitudinal studies involving cancer patients. Despite lengthy and labor-intensive, such studies will certainly provide answers for currently unsolved questions in cancer research.

At this point, it is clear that EVs have a tremendous potential to be used as a “liquid biopsy” for cancer patients, which would be less invasive compared to surgery and may provide diagnostic information, aid in therapeutic decisions, and monitoring of disease over time, on a personalized basis.

## Conflict of Interest Statement

The authors declare that the research was conducted in the absence of any commercial or financial relationships that could be construed as a potential conflict of interest.
